# Food Ingredients and Nutraceuticals from Microalgae: Main Product Classes and Biotechnological Production

**DOI:** 10.3390/foods10071626

**Published:** 2021-07-14

**Authors:** Regina Kratzer, Michael Murkovic

**Affiliations:** 1 Institute of Biotechnology and Biochemical Engineering, Graz University of Technology, NAWI Graz, Petersgasse 10-12/I, 8010 Graz, Austria; regina.kratzer@tugraz.at; 2 Institute of Biochemistry, Graz University of Technology, NAWI Graz, Petersgasse 10-12/II, 8010 Graz, Austria

**Keywords:** food ingredient, microalgal pigments, n-3-fatty acids, microalgae cultivation, growth conditions, cultivation systems

## Abstract

Microalgal products are an emerging class of food, feed, and nutraceuticals. They include dewatered or dried biomass, isolated pigments, and extracted fat. The oil, protein, and antioxidant-rich microalgal biomass is used as a feed and food supplement formulated as pastes, powders, tablets, capsules, or flakes designed for daily use. Pigments such as astaxanthin (red), lutein (yellow), chlorophyll (green), or phycocyanin (bright blue) are natural food dyes used as isolated pigments or pigment-rich biomass. Algal fat extracted from certain marine microalgae represents a vegetarian source of n-3-fatty acids (eicosapentaenoic acid (EPA), docosahexaenoic acid (DHA), γ-linolenic acid (GLA)). Gaining an overview of the production of microalgal products is a time-consuming task. Here, requirements and options of microalgae cultivation are summarized in a concise manner, including light and nutrient requirements, growth conditions, and cultivation systems. The rentability of microalgal products remains the major obstacle in industrial application. Key challenges are the high costs of commercial-scale cultivation, harvesting (and dewatering), and product quality assurance (toxin analysis). High-value food ingredients are commonly regarded as profitable despite significant capital expenditures and energy inputs. Improvements in capital and operational costs shall enable economic production of low-value food products going down to fishmeal replacement in the future economy.

## 1. Introduction

Microalgae are microscopic photosynthetic organisms that are found in marine, as well as in freshwater, environments. These unicellular organisms have a size ranging from a few to several hundred micrometers, depending on class and species. Most microalgae have photosynthetic mechanisms similar to land-based plants but generally more efficient in the photoautotrophic conversion of solar energy into biomass. This is since the cellular structure of microalgae is less sophisticated, and the normal environment is aqueous with easy access to CO_2_ and further nutrients [1]. Industrially significant organisms are prokaryotic cyanobacteria, as well as eukaryotic microalgae that include diatoms, golden microalgae, and some green microalgae species. Cyanobacteria (Cyanophyceae or blue-green algae) include the well-known *Spirulina* species *Arthrospira platensis* and *Arthrospira maxima* (aka *Spirulina platensis* and *Spirulina maxima*). Blue-green algae are found in a variety of habitats. Diatoms are a major group of microalgae. Living in the oceans, the diatoms make up a significant portion of the biomass on Earth. They often accumulate oils and chrysolaminarin (a storage polysaccharide). Green algae are especially abundant in freshwater. The main storage compound of green algae is starch, although oils can be produced as well. The freshwater green algae *Haematococcus pluvialis* is commercially important as a source of astaxanthin, *Chlorella vulgaris* as a supplementary food product or food ingredient, and the halophilic algae *Dunaliella salina* as a source of β-carotene. The golden algae are known for producing oils and carbohydrates [2]. Some prokaryotic and eukaryotic microalgae are known for the production of toxins that can contaminate the environment as well as algal cultures [3]. The chemical structures of the toxins comprise peptides (e.g., microcystin), alkaloids (e.g., saxitoxin), and alkyl phenols (e.g., aplysiatoxin).

Microalgae are a resource for food and feed with high potential. Currently, the most cultured species are *Arthrospira platensis* and *Arthrospira maxima, Chlorella vulgaris*, *Dunaliella salina*, and *Haematococcus pluvialis*, which can be grown photoautotrophically with sunlight as an energy source. The carbon source is CO_2_, which can be supplied by gassing or supplementing the water with soluble carbonates. Heterotrophic cultivation on organic carbon sources such as sugars is used for the production of n-3 fatty acids with marine organisms (*Crypthecodinium cohnii, Schizochytrium* sp. TC 002, and Ulkenia sp. strain TC 010) in fermenters excluding sunlight [4]. For feed for fish aquaculture, the main cultivated species are: *Chlorella vulgaris*, *Isochrysis galbana*, *Pavlova* sp., *Phaeodactylum* *tricornutum*, *Chaetoceros* sp., *Nannochloropsis oceanica*, *Skeletonema* sp., *Thalassiosira* sp., *Haematococcus pluvialis*, and *Tetraselmis suecica* [2,5,6]. However, the technology to produce microalgae is still immature. Research and development have been done in recent years and continue on cultivation systems. A leap in the development of microalgae technology is required; on a practical level, the scale of production needs to increase with a concomitant decrease in the cost of production [1]. Microalgae are cultivated in a wide range of different cultivation systems that can be placed outdoors or indoors. Cultivation systems range from open shallow raceway ponds to closed photobioreactors. The systems mostly used on a large scale and on a commercial basis are open systems [7]. For human consumption, the microalgae *Arthrospira platensis* and *Arthrospira maxima* are the most common and most intensively investigated organism. In nature, these microalgae grow in specific lakes with high pH around the world. As these microorganisms contain gas, they are floating on the water and can be harvested easily from the surface, as is done at lake Texcoco (Mexico), Lake Chad, and Lake Kossorom (both in Chad) [1].

The amount of literature covering microalgae production and use in foods (and feed) is overwhelming. Therefore, the scope of the present review is to give an overview of microalgae production in a concise manner, including cultivation (growth conditions, cultivation systems, limitations) and downstream processing. Because of its nutritionally excellent composition (amino acids, vitamins, lipids), the microalgae are eaten as is, are included in traditional foods as ingredients, or as food additives (colorants).

## 2. Microalgae Production

A literature search using the keywords “microalgae cultivation” results in tens of thousands of hits, with approximately half of the articles published in the last five years. Therefore, and despite dozens of comprehensive review articles, gaining an overview remains a time-consuming task. Here, we strive to cover the necessary considerations in microalgae cultivation in a concise manner, including illumination and further growth conditions, cultivation systems, microbial contaminations, and downstream processing.

### 2.1. Microalgae Cultivation

#### 2.1.1. Illumination

Low cell densities and moderate growth rates are the major obstacles towards a broad commercial use of microalgal products. The most important cultivation parameters are light and nutrient supply. Decisive factors of light supply are light intensity, spectral range, and photoperiod. Optimal light supply is case-dependent with regard to the type of microalgae but also to the type of product. Generally, the rate of photosynthesis correlates with light supply until photo-inhibition is reached. Excessive illumination causes damage to the photoreceptor system leading finally to cell death [7,8]. Therefore, photosynthetic organisms contain pigments for light-harvesting and photoprotection. Light-harvesting pigments such as chlorophylls, phycobilins, several carotenes, and xanthophylls are structural and functional components of the photosynthetic apparatus. Photoprotective pigments are astaxanthin, β-carotene, and other carotenoids. Production of light-harvesting pigments is supported by subsaturating light conditions (photoacclimation; pigment concentrations typically 0.5 g/g_CDW_). Photoprotective pigments are overproduced in response to environmental stress, such as oversaturating light intensity, high salt concentration, or nitrogen limitation (pigment concentrations up to 70 mg/g_CDW_) [9,10].

#### 2.1.2. Diverse Growth Conditions

**Figure 1:**
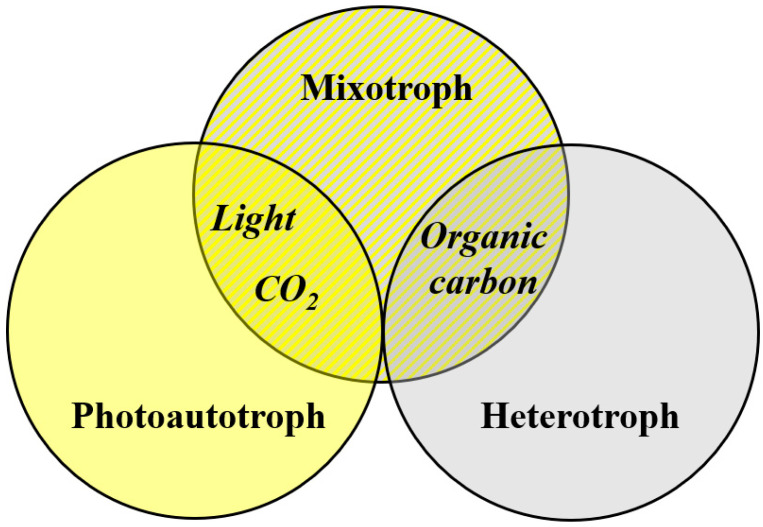
Energy and carbon sources of microalgae.

Microalgae show diverse metabolic pathways. Other than the dominant photoautotrophy, secondary heterotrophy and mixotrophy is encountered (Figure 1). All microalgae are photoautotrophs, with autotrophic cultivation being the main method for biomass production [11]. Photoautotrophs use light as an energy source to fix carbon via photosynthesis. Large-scale, photoautotrophic cultivation is mostly realized in open systems. Strains that tolerate/require high salinity or alkaline pH are used so that cultures are protected from contaminants. Prominent examples are *Arthrospira platensis* and *Dunaliella salina* [12]. Generally, lower biomass concentrations are obtained with microalgae cultivated photoautotrophically compared to mixotrophic and heterotrophic cultivation strategies [13]. Microalgae capable of mixotrophic growth can metabolize CO_2_ and light simultaneously with organic carbon sources but can also switch to sole phototrophic or sole heterotrophic growth. These microalgae show higher growth rates under mixotrophic conditions as compared to sole heterotrophic or photoautotrophic conditions [14]. A prominent example of mixotrophic cultivation is astaxanthin production by *H. pluvialis* (e.g., [15]). Generally, mixotrophic growth requires less light compared to photoautotrophic growth. Heterotrophs can grow on organic carbon in the absence of light (no illumination required). Again, growth rates under heterotrophic conditions are higher than those under photoautotrophic conditions. However, other microorganisms compete with heterotrophic microalgae for the carbon source, and sterile conditions are required to avoid culture contamination and product loss [12,13]. 

### 2.2. Cultivation Systems

Key considerations in the set-up of algae cultivation are the decisions between open ponds (mostly outdoor) or closed photobioreactors (mostly indoor). A simple characterization of the main cultivation systems is given in Table 1. 

#### 2.2.1. Open Ponds 

**Figure 2:**
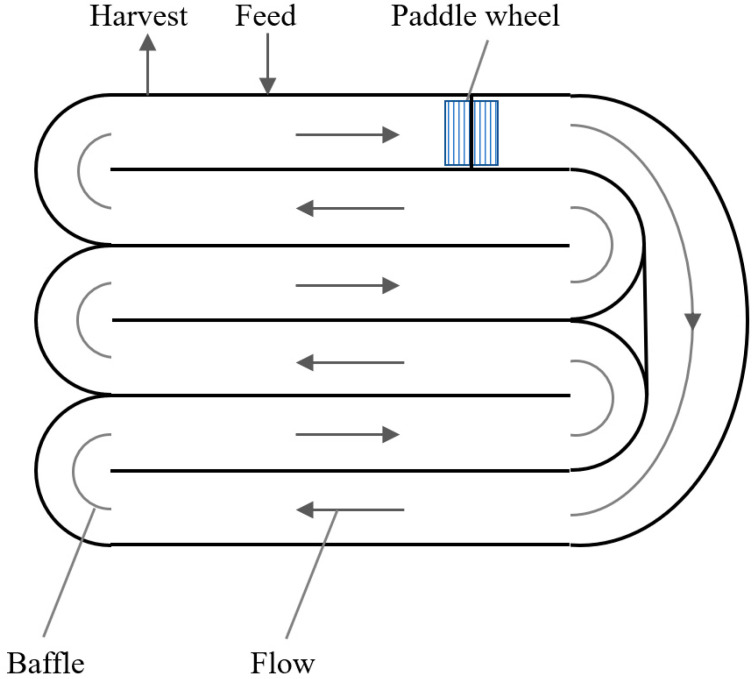
Scheme of a raceway pond.

Classical algae cultivation uses open ponds in natural habitats or shallow artificial ponds often mixed by a rotating arm or by paddle wheels (Figure 2). Mixed, open ponds, i.e., raceway-ponds or circulating-open-ponds, are characterized by low power requirements, operating costs, and capital costs. However, open-pond cultivation is prone to risks of contamination and other pollution, low possibilities of parameter control (especially illumination), loss of water by evaporation, and high weather dependence. The main limitations stem from insufficient mixing, causing a suboptimal supply of CO_2_ and restricted illumination. Cultivation times are approximately 6–8 weeks, with cell densities of about 0.1 or 25 g/m^2^d. Furthermore, weather-dependent cultivation parameters and unsterile cultivation conditions lead to low reproducibility [7]. The use of controlled, closed photobioreactor systems (PBRs) results in higher biomass (and product concentrations) and reproducible results. A 13-fold higher volumetric biomass productivity of PBRs compared to raceway ponds in the cultivation of microalgae for biodiesel production was reported by Chisti [16].

#### 2.2.2. Closed Photobioreactors 

**Figure 3:**
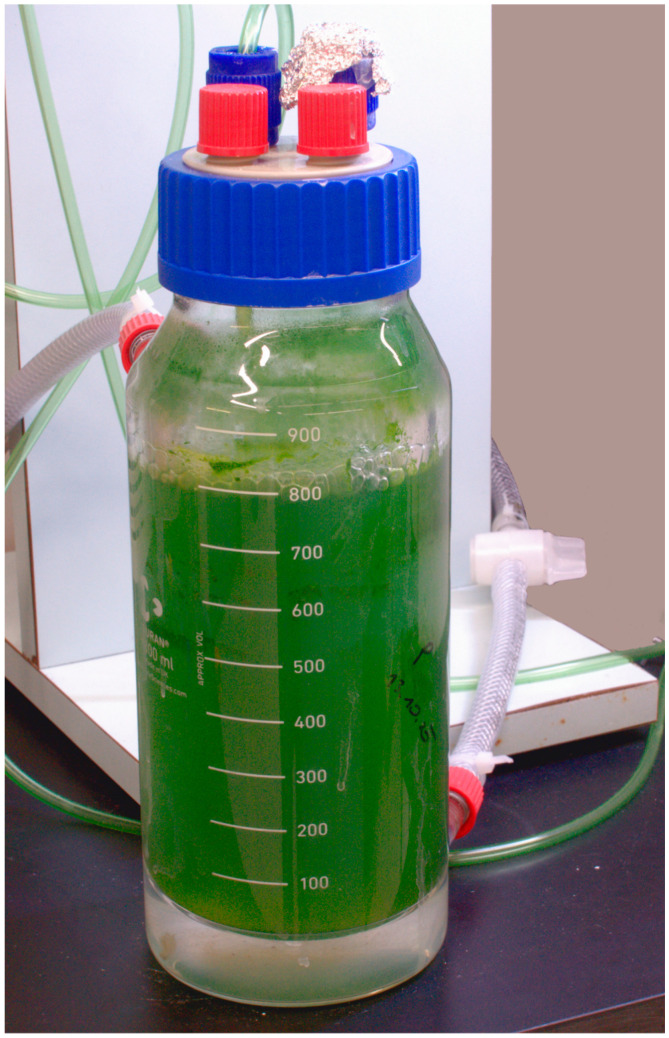
Culture of *Arthrospira platensis* in a bubble column type bioreactor. Slight foam formation and cell growth on the vessel walls were experienced (photo: M. Murkovic).

PBRs facilitate increased biomass and product concentrations. PBRs protect the algal culture against (microbial) contaminations, provide nutrients, illumination, pH and temperature control, and outgassing of the produced oxygen. Mixing and illumination are again the most important parameters. Optimal cultivation conditions provide gentle mixing so that cells, carbon dioxide, and other essential nutrients are evenly circulated. Cell movement facilitates constant illumination and prevents cell growth on vessel walls (see also Figure 3). The simplest PBRs consist of transparent plastic bags or plastic columns operated as bubble columns and airlift reactors (also referred to as vertical tubular reactors) (Figure 3). The systems are mixed by the bubbling of CO_2_-enriched air from the bottom. Stirred tank reactor-type (STR) PBRs, such as ones used for bacteria and yeast production, provide the most efficient mixing. Many microalgae strains are, however, sensitive to shear forces. Horizontal tubular reactors (HTRs), made from glass or plastic tubes, have succeeded within the production scale [17]. Aeration is accomplished in the tube connections and illumination over the length of the tube [8,18]. Clearly, microalgae cultivation in closed bioreactors is more energy-intensive compared to open ponds [19]. 

### 2.3. Contaminations

A major bottleneck in the industrial production of products from algae is that algae cultures are prone to contaminations. To avoid contamination, closed systems can be used; however, in open systems (large scale), contamination is unavoidable. These are either organisms that graze upon and feed on the algae or undesired algae strains that take over the cultivation media. Contaminations can lead to culture crashes and product loss, and also the formation of toxins [20,21]. Therefore, effective strategies to avoid, repress, monitor, and control contaminants without interfering with the growth of the target microalgae are required. Grazers and contaminants experienced (especially in open-pond cultivations) are mainly insect larvae, protozoans (ciliates, amoeba, dinoflagellates), viruses, fungi, bacteria, and other algae strains [22]. Selective growth conditions are effective measures against grazers and contaminants. The strict photoautotrophs *Arthrospira platensis* and *Dunaliella salina*, two of the most often cultivated microalgae, can grow in extreme environments. *A. platensis* tolerates high bicarbonate concentrations (0.2 M) and pH values of up to 10.2, whereas halophilic *Dunaliella salina* tolerates NaCl concentrations up to 4.0 M [23]. *Haematococcus pluvialis* cultures that are grown under mixotrophic conditions for astaxanthin production are much more susceptible to contaminations. Wen and co-workers [15] described a successful strategy to cultivate *Haematococcus pluvialis* under mixotrophic conditions in an outdoor raceway pond. *Haematococcus pluvialis* cultivation started under photoautotrophic conditions until nitrate is depleted from the medium, and then the culture is supplemented with acetate/acetic acid. Under mixotrophic conditions, *Haematococcus pluvialis* cells grow intracellular nitrogen pool, while bacterial reproduction is limited. Further measures against grazers and contaminants include the addition of chemicals (hypochlorite, pH shocks, ozone), addition of pesticides, or herbicides [15,24,25]. Another approach is the co-cultivation of microalgae to obtain a more robust polyculture compared to monocultures [26].

### 2.4. Downstream Processing

Similar to the majority of bioprocesses, downstream processing constitutes a considerable cost factor. Specifically, the cost for the harvest of microalgae was calculated as 20–30% of the total production cost of cheap bulk products such as biodiesel [27]. The main problems in microalgae separation are small cell sizes (2 to 30 µm), slow gravity settling (<10^−5^ m s^−1^), and low cell densities [28]. Harvesting requires one or more liquid-solid separation steps such as coagulation, flocculation, sedimentation, flotation, filtration, and centrifugation. Most microalgae cells do not aggregate spontaneously (an exception is *Anabaena* sp. ATCC 33047) [29]. Cell-cell contact is prevented by repulsion due to negatively charged cell surfaces. A change of pH or electrolyte addition reduces surface charges, facilitate close cell-cell contact resulting in van der Waals attraction and coagulation. Similarly, cationic polymers screen negative charges, bridge cells, and lead to flocculation. Gravity settling (sedimentation) depends on cell diameter and density differences between cells and medium. Settling velocities of single cells are low (<10^−5^ m s^−1^). Therefore, coagulation, flocculation, and flotation are frequently used to accelerate sedimentation (~10^−4^ m s^−1^). During flotation, rising air bubbles (10 to 100 µm) attach to cells and aid in collecting cells at the liquid surface [28,30]. Flocculated cells can be separated by macrofiltration membranes (pore sizes >10 µm) with the advantage of a lower energy requirement (i.e., lower pressure). In a previous study, crossflow ultrafiltration and microfiltration for the concentration of *Dunaliella salina* cells were compared. Frequent backflushing to alleviate membrane fouling (intrapore fouling and cake formation/pore-blocking) was required to maintain the transmembrane flow. Interestingly, average permeate fluxes and cell integrity losses were similar in the two systems. Rapid drops in transmembrane fluxes with the microfiltration membrane were ascribed to severe intrapore fouling. A clearer permeate was obtained with ultrafiltration [31]. Microalgae harvesting by centrifugation requires high capital investment and energy costs and is frequently used in the manufacturing of high-value products. Economic considerations require preconcentration steps in the industrial production of medium and low-cost products. 

In the majority of applications, subsequent energy-intensive dewatering and biomass drying steps are required. Discussion of further downstream processing is highly strain and product-specific. Extraction of oils and dyes requires cell rupture, centrifugation, and/or solvent extraction [31]. The conditions of cultivation, harvesting, and subsequent processing clearly influence the nutritional composition of microalgae. For example, protein loss by the drying of *Arthrospira platensis* was previously reported. With freeze-drying, 5% of protein was lost, whereas spray, convective, and infrared drying led to protein losses of 10 up to 25% [32].

## 3. Rentability of Microalgae Production

One of the major concerns relates to the rentability of microalgal products. Key challenges are the high production costs of products made up of commercial-scale cultivation, harvesting (dewatering), and finally, product quality assurance. The value of microalgal products spans from exceptionally high market values (e.g., EPA 4600 USD/kg) to products in the middle price segment (e.g., biomass for food and feed 10 to 50 USD/kg) down to fishmeal replacement (fishmeal price is approximately 2 USD/kg) [33]. Several algal-based foods have been launched in the last years: Microalgae biomass is used in the form of powders, tablets, and capsules. Especially in Western countries, the number of snacks and drinks containing microalgae doubled in the past few years. Most new products launched were bakery, meals, and chocolate confectionery [34]. Improvements in capital and operational costs shall enable economic production of low-value food and feed products going down to fishmeal replacement in the future economy [33,35]. 

## 4. Nutritional Composition of Microalgae

The composition of the microalgae is similar to other bacteria. Usually, they contain high amounts of protein, with all essential amino acids present. Especially the microalgal species *Arthrospira platensis*, *Chlorella vulgaris*, *Dunaliella salina*, *Haematococcus pluvialis*, and *Scenedesmus obliquus* show a very high content of protein with values higher than soybean, corn, and wheat (Table 2). Polyunsaturated fatty acids are occurring in significant amounts, including linoleic acid, γ-linolenic acid (GLA), eicosapentaenoic acid (EPA), docosahexaenoic acid (DHA), and arachidonic acid (Table 3). Generally, microalgae show a high protein content when cultured in media with high concentrations of nitrogen source and under well-balanced growth conditions. The accumulation of lipids and carbohydrates is induced under stress conditions. (Note that green algae show a nitrogen requirement that corresponds to 5–10% of their biomass. Culture media developed for microalgae cultivation contain between 5 and 50 mM to avoid nitrogen limitation) [36]. In addition, a series of water-soluble vitamins are present. Bioactive vitamin B_12_ (cobalamin) is normally not present in microalgae as it is synthesized as pseudocobalamin in planktonic cyanobacteria [37]. A series of minerals, including iodine, is also occurring [38]. Although iodine can occur in higher concentrations, it rarely poses a health problem, acting mainly by interfering with the thyroid gland metabolism resulting in thyrotoxicosis [39,40]. A series of pigments are also available that are involved in photosynthesis. These comprise chlorophyll a, a series of carotenoids (e.g., β-carotene, zeaxanthin, β-cryptoxanthin) and, in *Arthrospira platensis*, the blue pigments c-phycocyanin and allo-phycocyanin [41]. Other than being a valuable source of macronutrients, the microalgae can also contain polyunsaturated fatty acids. Of special interest are the essential fatty acids comprising linoleic acid and the n-3 fatty acids. Dolganyuk and co-workers studied the lipid complex of different microalgae (*Chlorella vulgaris*, *Dunaliella salina*, *Arthrospira platensis*). They found a huge variation in the fatty acid composition, especially in the n-3 to n-6 ratio, with *Dunaliella salina* containing the highest amount of n-3 fatty acids (ALA, DHA) [2]. 

The analyses of the protein composition have shown that, for most of the microalgal species (except *Aphanizomenon* sp.), the eight essential amino acids occur in equal or higher amounts than recommended by the WHO/FAO [44]. The quality of the proteins with respect to biological value, digestibility, utilization, and protein efficiency ratio is equal to conventional plant proteins [45]. 

Sandgruber and co-workers published the compositional analyses of a series of commercially available dried microalgal products [46]. The concentrations of the minerals (Ca, Mg) and the trace elements (Fe, Mn, Ni, Cu, Zn, Mo, Se, I) showed a high variation between the species. Even when analyzing different products of the same species, a high variation was observed. This is especially the case with calcium, magnesium, and zinc having a variation of two to three orders of magnitude. A similar situation was observed with the heavy metals As, Cd, Hg, and Pb.

## 5. Food Ingredients and Nutraceuticals from Microalgae

### 5.1. Microalgae Used as a Food Ingredient

When using microalgae as a food ingredient, some limitations are obvious. This is primarily because of the intensive green color that is added to the recipes and a fishy aroma [47]. As the flavor is one of the biggest disadvantages of algae-containing food, a lot of work was invested in masking this flavor by using intensive spices [48] or by using the aroma of the microalgae to bring the typical aroma of seafood out. A commercial product [49,50] was developed that shows microalgae can be used as an ingredient in foods with a typical seafood aroma. Many products from algae are commercialized directly as biomass. These are sold as nutritional supplements since the microalgae, mostly *Arthrospira platensis*, contain high amounts of protein and n-3 fatty acids. Other products derived from *Haematococcus pluvialis* are used for supplementing the carotenoids astaxanthin and lutein. Since microalgae are also known for having high concentrations of n-3 fatty acids (mainly *Chlorella vulgaris* and *Arthrospira platensis* containing γ-linolenic acid, eicosapentaenoic acid, docosapentaenoic acid, docosahexaenoic acid, Table 2), extracts are developed that might be used to upgrade foods with these fatty acids.

#### 5.1.1. Addition of Microalgae to Biscuits

Spray-dried biomass of *Arthrospira maxima* can be used as an ingredient for biscuits that can improve the nutritional quality [48]. In their experiments, da Silva and co-workers could show that the content of iron was improved in parallel to the protein and lipid content. In addition, the products were enriched with valuable amino acids and polyunsaturated fatty acids with high amounts of γ-linolenic acid (n-6 fatty acid). It is interesting to note that the sensory profile of the biscuits was acceptable to the consumers with a limitation related to the color. Up to an addition of 20% of spray-dried biomass, the sensory score was acceptable. In this case, the *Arthrospira maxima* biomass was spray-dried in the presence of starch sodium octenyl succinate (E 1450, emulsifier, foaming agent). The good sensory properties of the final biscuits were attributed to the presence of the food additive, which might mask the typical algal aroma. The addition of microalgal biomass to modify the texture of foods is another possibility to include the cyanobacteria [51]. The idea behind the concept of Bernaerts and co-workers was to use the biopolymers that are occurring in microalgae to change the viscosity of the food product into which the microorganisms are integrated. These polymers comprise the intracellular proteins and starch-like structures that are liberated by the disintegration of the cells as well as the cell wall polysaccharides and exopolysaccharides. However, the diversity of the polysaccharides is huge, and it is difficult to predict the composition and relate it to the chemical properties. Due to this diversity of structures and compositions, there is limited knowledge of the rheological properties and potential applications for foods [42]. In addition to these biopolymers, resistant polymers can be present in some species (e.g., *Haematococcus pluvialis*, *Chlorella* sp., *Scenedesmus* sp., *Nannochloropsis* sp.). These are called algaenans, being polymers of carbon chains that are cross-linked by ether and ester bonds. Sahni and co-workers [52] investigated if the remaining biomass after extraction of chlorophyll could be used as an ingredient in the preparation of cookies. They studied how much of the wheat flour could be replaced by the microalgal meal that originated from *Chlorella* sp. (Abca-17). Some of the textural parameters changed with the addition of 1–12% of the microalgal meal, i.e., the weight and thickness increased, whereas the diameter, spread ratio, and spread factor decreased with increasing content. By adding up to 6% microalgal powder, no significant difference was found in a sensory evaluation. A change in the mouthfeel was also observed, meaning that the necessary force for breaking the fortified cookies increased in parallel with the addition. The observed color changes were a darkening and increased greenness in the cookies with increasing content of the algal biomass. The addition of *Arthrospira platensis* powder to a standard recipe for cookies with concentrations of up to 3% resulted in an increase of hardness and lower sensory scores. The color change was obvious even at the lowest tested level of algae addition of 1% [53]. 

#### 5.1.2. Addition of Microalgae to Pasta and Noodles

Flours obtained from *Chlorella vulgaris* and *Arthrospira platensis* in combination with the macroalgae *Eucheuma cottonii* were added to wheat flour to produce noodles. It was shown that when adding the algal biomass to the dough of the noodle, the content of protein, fat, ash, and dietary fiber increased, whereas the carbohydrate content reduced with no changes in moisture. These changes are due to a new composition of the ingredients. The therewith produced noodles were shown to have acceptable culinary properties, such as texture, color, aroma, and flavor. As an example, the optimal composition of the flour consisted of 90 g wheat flour, 5 g *Arthrospira platensis*, and 5 g *Eucheuma cottonii* dried biomass resulting in noodles with a low fat and high protein content [54]. Since pasta is a staple food consumed worldwide, it might be an excellent target for improving the nutritional status by adding high-value ingredients. When developing recipes with new ingredients that improve the nutritional value, it has to be considered that not only the texture and cooking properties might change but also the sensory properties. De Marco and co-workers [55] could show that up to 20 g of *Arthrospira platensis* powder could be added per 100 g of flour with a result of a technologically acceptable product. Similar results were obtained by the group of El-Baz [56], who investigated the addition of a powder of the microalgae *Dunaliella salina* to pasta preparation. The sensory evaluation of the pasta was changing with a microalgae content of more than 2%. Up to 2%, the mouthfeel and overall acceptability was not changed. The color changed to green, which is a result of the chlorophyll being introduced with the microalgae. A comparable experiment was carried out using *Chlorella vulgaris* and *Arthrospira maxima* as an ingredient for pasta. The addition of algae was up to 2% resulting in products with different colors. Green (chlorophyll) or orange (cantaxanthin) for pasta with added chlorella and green with a blue shade (probably because of the presence of blue phycocyanin) for pasta with *Arthrospira platensis*. The texture improved with increasing amounts of integrated algal biomass, which might have been due to the higher content of protein and lower water uptake. However, the products with higher contents of algae had a lower sensory score [57]. 

### 5.2. Isolated Products from Microalgae for Enrichment of Foods

#### 5.2.1. n-3 Fatty Acids

Some of the cyanobacteria are known for their high content of n-3 fatty acids (Table 3). In addition to these, a series of species are known for containing EPA and DHA in the range of 17–45% of the total lipids (e.g., *Nannochloropsis oceanica* and *Nannochloropsis salina*, *Pinguiococcus pyrenoidosus*, *Thraustochytrium* sp., *Chlorella minutissima*, *Dunaliella salina*, *Pavlova viridis* and *Pavlova lutheri*, *Isochrysis galbana*, *Schizochytrium* sp., *Crypthecodinium cohnii*, *Aurantiochytrium* sp., *Phaeodactylum tricornutum*) [58]. Since the alimentary supply of n-3 fatty acids is generally limited due to limited availability of fish oils, a series of efforts are made to increase the content of these fatty acids in foods. One of the possibilities is to enrich food products with cyanobacteria containing high amounts of highly unsaturated fatty acids [59]. Adding microalgae to pasta, in general, can improve the nutrient composition, as discussed earlier. The main problem associated with highly unsaturated fatty acids is their low oxidative stability, since deteriorated products produce a rancid aroma in addition to the health problems related to the uptake of oxidized oils [60]. However, from a nutritional point of view, the uptake of n-3 fatty acids from microalgal oil is comparable to fish oil with the possible advantage of a better supply with carotenoids [61]. In addition to adding the n-3 fatty acids directly to the food, it is possible to enrich animal products by supplying the unsaturated fatty acids with the feed. This was shown, for example, by Lemahieu and a co-worker [62], who could increase the DHA content of eggs when feeding *Isochrysis galbana* to the chicken. Polyunsaturated fatty acids (PUFAs), particularly the n-3 fatty acids having five (EPA) or six (DHA double bonds, are prone to oxidation due to the lability of the hydrogens linked to the methylene groups between the double bonds. These PUFAs require stabilization against oxidation. Shen and co-workers could show that a combination of octyl gallate with tea polyphenol palmitate showed the most effective protection [63].

#### 5.2.2. Carotene and Other Carotenoids

Microalgae are known for producing high amounts of carotenoids. *Dunaliella salina* can produce β-carotene up to 13% of dry weight. Astaxanthin can be found in *Haematococcus pluvialis*, which produces up to 7% of the dry weight. Canthaxanthin was found in *Coalstrella striolata* containing close to 5% of dry weight. Lutein, zeaxanthin, fucoxanthin, echinenone, and violaxanthin could also be identified in different species [64]. The EFSA Panel on Nutrition, Novel Foods, and Food Allergens concluded that an intake of 8 mg of astaxanthin from food supplements is safe for adults. This is also true for the combination with the background diet [65]. The evaluation of mixed carotenes and β-carotene of different sources showed that no acceptable daily intake (ADI) could be established for use as a food color. However, the total uptake of the carotenoids should be in the range of 5–10 mg/day. This includes the safe exposure to β-carotene of below 15 mg/day, which does not increase the cancer risk [66]. A long list of bioactivities of the algal carotenoids that are related to human health was established [67]. In addition to the well-described vitamin A activity and reduction of age-related macular degradation (AMD) by lutein [68], these compounds can have an impact on human health, comprising anti-cancer, anti-obesity, anti-inflammatory, antidiabetic, antimicrobial, and neuroprotective activities.

### 5.3. Microalgae as Food Supplements

As mentioned earlier, the nutritional composition of microalgae is of high quality with respect to amino acids, fatty acids, and minor components such as vitamins and pigments. With these high concentrations of nutrients, microalgae are predestined for the manufacturing of food supplements. The main microalgae that are marketed as dried powder, capsules, or pressed pills are *Chlorella vulgaris* and *Arthrospira platensis.* A series of positive health effects of microalgae are described ranging from an influence on the cholesterol level and associated coronary heart diseases, antiviral, antimicrobial, and antifungal activity. The presence of sterols (e.g., clionasterol) implies all the potential positive health effects that are related to an intake of these compounds [69]. *Arthrospira platensis* is especially known for its high content of γ-linolenic acid, one of the essential fatty acids. It is also rich in water-soluble vitamins. In addition to the effects of *Arthrospira platensis*, *Chlorella vulgaris* has been characterized by the presence of dietary antioxidants such as lutein, α- and β-carotene, and vitamins C and E. In *C. vulgaris* the most interesting compound is β-1,3-glucan, which might stimulate the human immune system [70]. Astaxanthin, another carotenoid with beneficial health effects, is found in high concentrations in *Haematococcus pluvialis* [71]. It was approved as a food supplement by the EFSA in 2020 [65].

### 5.4. Toxicological Relevance of Microalgal Products

Some of the cyanobacteria species are known for their potential of producing potent toxins. When these microalgae contaminate the cultures for food production, the toxins pose a severe health risk. Some examples are, microcystin and related structures are produced by *Microcystis aeruginosa*, saxitoxin by *Aphanizomenon flos-aquae*, and anatoxins by *Anabaena flos-aquae*. The chemical structures comprise cyclic peptides (microcystin), alkaloids (anatoxin, saxitoxin), and lipopolysaccharides affecting the liver, nerve axons, and synapse, as well as the gastrointestinal tract [3]. The group of Dietrich published an overview on the presence of algal toxins in commercially available food supplements. In their study, they only found microcystins, which were present in products of *Aphanizomenon flos-aquae*. Extracts from all the tested samples showed cytotoxic effects, which leads to the conclusion that additional components should be present that have the potential to induce adverse effects in consumers [72].

## 6. Conclusions

**Past.** Microalgae have been consumed as a food for a long time: Spanish conquerors reported that Aztecs consumed cyanobacteria *Arthrospira platensis* and *Arthrospira maxima* as a dry cake. In Chad, *Arthrospira platensis* has been used as food on a daily basis for centuries. The filamentous cyanobacteria *Nostoc commune*, *Nostoc flagelliforme*, and *Nostoc punctiforme* are traditionally consumed in China, Mongolia, Tartaria, and South America. In Japan, the cyanobacterium *Aphanotheca sacrum* is a traditional delicacy (reviewed in [73]).

**Present**. Microalgae-based products for food (and feed) contain either whole dried biomass (*Arthrospira platensis*, *Chlorella vulgaris*) or isolated components (astaxanthin, β-carotene, phycocyanin, and the n-3 fatty acids EPA and DHA).

Recently a comprehensive list of commercially available products from microalgae and products containing microalgae as an ingredient was published by Eltanahy and Torky [74].

The steadily increasing interest in the use of microalgae in the food industry is based on two major developments:

- New hygienic cultivation systems for the production of pure cultures under controlled conditions to achieve high cell densities. High product concentrations (i.e., biomass, fat, secondary metabolites) are a prerequisite for industrial production. Improved cultivation systems facilitate the industrial production of selected species (*Arthrospira platensis*, *Chlorella vulgaris*) with high nutritional values (high protein contents) and interesting metabolites (food colorants, antioxidants etc.).
- Consumers demand vegan foods, natural foods, and sustainable food production. Microalgae has become a ‘fashion nutraceutical’ in Western countries.

**Future**. Microalgal biomass is considered by scientists, engineers, and investors, along with insects and mycoprotein, as a future food to mitigate malnutrition. Future foods are characterized by high protein content, low requirement for chemicals (fertilizers, hormones etc.), and a low greenhouse gas footprint [1].

The authors expect that in the near future, (i) the production price of microalgae will decrease, (ii) microalgae will be cultivated for the sake of protein production, and (iii) a series of newly developed products from microalgae will enter the food market.

## Figures and Tables

**Table 1:** Summary of microalgae cultivation strategies with regard to the main characteristics (STR PBR: stirred tank reactor photobioreactor).

*Parameter*	Open Ponds	Raceway Ponds	Bubble Columns	Tube Reactors	STR PBR
*Mixing*	very low	low	medium	medium	high
*Illumination*	very low	low	medium	high	medium
*Sterility*	no	low	possible	possible	yes
*Capital and operational cost*	very low	very low	medium	medium	high

**Table 2:** Macronutrient composition of commercially important microalgae (extracted from [1,17,42,43].

*Species*	Protein (wt%)	Carbohydrate (wt%)	Lipid (wt%)
*Nannochloropsis* sp.	29–32	9–36	15–18
*Nannochloropsis oceanica*	29	32–39	19–24
*Botryococcus braunii*	70	–	–
*Arthrospira platensis*	53–70	12–24	6–20
*Chlorella vulgaris*	49–55	7–42	3–36
*Haematococcus pluvialis*	48	27	15
*Isochrysis galbana*	27	17	17
*Dunaliella salina*	57	32	6
*Scenedesmus obliquus*	50–56	10–17	12–14
*Porphyridium cruentum*	28–39	40–57	9–14

**Table 3:** Polyunsaturated fatty acids in microalgae (extracted from [17,43]; given in % of total fatty acids).

*Species*	LA	ALA	GLA	STA	AA	EPA	DPA	DHA
*Chlorella vulgaris*	3–10	2–21	15–24	Traces	Traces	3	3	21
*Arthrospira platensis*	10–21	1	1–25	1	0.3–0.4	2–3	-	3
*Isochrysis galbana*	1	0.5	0.5	1	1	2	Traces	19
*Scenedesmus obliquus*	1–2	4–22	0.1–4	1–3	0.1–0.2	-	-	-
*Haematococcus pluvialis*	4	15	0.1	-	-	-	-	-
*Porphyridium cruentum*	0.4–25	-	-	-	1–35	1–27	-	6.1
*Nannochloropsis oceanica*	0.6–0.8	-	-	-	1.4–1.9	8.4–11	-	-

LA: linoleic acid, ALA: α-linolenic acid, GLA: γ-linolenic acid, STA: 18:4n-3 stearidonic acid, AA: 20:4n-6 arachidonic acid, EPA: 20:5n-3 eicosapentaenoic acid, DPA: 22:5n-3 docosapentaenoic acid, DHA: 22:6n-3 docosahexaenoic acid.

## References

[1] Habib M.A.B., Parvin M., Huntington T.C., Hasan M.R. (2008). A Review on Culture, Production and Use of Spirulina as Food for Humans and Feeds for Domestic Animals and Fish.

[2] Dolganyuk V., Andreeva A., Budenkova E., Sukhikh S., Babich O., Ivanova S., Prosekov A., Ulrikh E. (2020). Study of morphological features and determination of the fatty acid composition of the microalgae lipid complex. Biomolecules.

[3] Katircioglu H., Akin B.S., Atici T. (2004). Microalgal toxin(s): Characteristics and importance. Afr. J. Biotechnol..

[4] Karnaouri A., Chalima A., Kalogiannis K.G., Varamogianni-Mamatsi D., Lappas A., Topakas E. (2020). Utilization of lignocellulosic biomass towards the production of omega-3 fatty acids by the heterotrophic marine microalga Crypthecodinium cohnii. Biores. Technol..

[5] Patil V., Källqvist T., Olsen E., Vogt G., Gislerød H.R. (2007). Fatty acid composition of 12 microalgae for possible use in aquaculture feed. Aquacult. Int..

[6] Bhattacharjya R., Marella T.K., Tiwari A., Saxena A., Singh P.K., Mishra B. (2020). Bioprospecting of marine diatoms Thalassiosira, Skeletonema and Chaetoceros for lipids and other value-added products. Biores. Technol..

[7] Koller M., Prokop A., Bajpai R.K., Zappi M.E. (2015). Design of closed photobioreactors for algal cultivation. Algal Biorefineries.

[8] Huang Q., Jiang F., Wang L., Yang C. (2017). Design of photobioreactors for mass cultivation of photosynthetic organisms. Engineering.

[9] Han D., Li Y., Hu Q. (2013). Astaxanthin in microalgae: Pathways, functions and biotechnological implications. Algae.

[10] Mulders K.J.M., Lamers P.P., Martens D.E., Wijffels R.H. (2014). Phototrophic pigment production with microalgae: Biological constraints and opportunities. J. Phycol..

[11] Morales-Sánchez D., Martinez-Rodriguez O.A., Martinez A. (2017). Heterotrophic cultivation of microalgae: Production of metabolites of commercial interest. J. Chem. Technol. Biotechnol..

[12] Hu J., Nagarajan D., Zhang Q., Chang J.-S., Lee D.J. (2018). Heterotrophic cultivation of microalgae for pigment production: A review. Biotechnol. Adv..

[13] Daneshvar E., Ok Y.S., Tavakoli S., Sarkar B., Shaheen S.M., Hong H., Luo Y., Rinklebe J., Song H., Bhatnagar A. (2021). Insight into upstream processing of microalgae: A review. Biores. Technol..

[14] Li T., Zheng Y., Yu L., Chen S. (2014). Mixotrophic cultivation of a Chlorella sorokiniana strain for enhanced biomass and lipid production. Biomass Bioenergy.

[15] Wen X., Wang Z., Ding Y., Geng Y., Li Y. (2020). Enhancing the production of astaxanthin by mixotrophic cultivation of Haematococcus pluvialis in open raceway ponds. Aquac. Int..

[16] Chisti Y. (2007). Biodiesel from microalgae. Biotechnol. Adv..

[17] Guerra I., Pereira H., Costa M., Silva J.T., Santos T., Varela J., Mateus M., Silva J. (2021). Operation regimes: A comparison based on Nannochloropsis oceanica biomass and lipid productivity. Energies.

[18] Carvalho A.P., Meireles L.A., Malcata F.X. (2006). Microalgal reactors: A review of enclosed system designs and performances. Biotechnol. Prog..

[19] Jones S.M.J., Harrison S.T.L. (2014). Aeration energy requirements for lipid production by *Scenedesmus* sp. in airlift bioreactors. Algal Res..

[20] Ger K.A., Urrutia-Corderob P., Frost P.C., Hansson L.-A., Sarnelle O., Wilson A.E., Lürling M. (2016). The interaction between cyanobacteria and zooplankton in a more eutrophic world. Harmful Algae.

[21] Jang M.-H., Ha K., Joo G.-J., Takamura N. (2003). Toxin production of cyanobacteria is increased by exposure to zooplankton. Freshw. Biol..

[22] Karuppasamy S., Musale A.S., Soni S., Bhadra B., Gujarathi N., Sundaram M., Sapre A., Dasgupta S., Kumar C. (2018). Integrated grazer management mediated by chemicals for sustainable cultivation of algae in open ponds. Algal Res..

[23] Richmond G., Richmond A., Hu Q. (2013). Biological principles of mass cultivation of photoautotrophic microalgae. Handbook of Microalgal Culture.

[24] Koller M., Salerno A., Tuffner P., Koinigg M., Böchzelt H., Schober S., Pieber S., Schnitzer H., Mittelbach M., Braunegg G. (2012). Characteristics and potential of micro algal cultivation strategies: A review. J. Clean Prod..

[25] Day J., Thomas J., Achilles-Day U.E., Leakey R.J. (2012). Early detection of protozoan grazers in algal biofuel cultures. Biores. Technol..

[26] Tejido-Nuñez Y., Aymerich E., Sancho L., Refardt D. (2020). Co-cultivation of microalgae in aquaculture water: Interactions, growth and nutrient removal efficiency at laboratory- and pilot-scale. Algal Res..

[27] Barros A.I., Gonçalves A.L., Simões M., Pires J.C.M. (2015). Harvesting techniques applied to microalgae: A review. Renew. Sustain. Energy Rev..

[28] Grima E.M., Fernández F.G.A., Robles-Medina A., Richmond A., Hu Q. (2013). Downstream processing of cell mass and products. Handbook of Microalgal Culture.

[29] González López C.V., Acién Fernández F.G., Fernández Sevilla J.M., Sánchez Fernández J.F., Cerón García M.C., Molina Grima E. (2009). Utilization of the cyanobacteria *Anabaena* sp. ATCC 33047 in CO2 removal processes. Biores. Technol..

[30] Koller M., Muhr A., Braunegg G. (2014). Microalgae as versatile cellular factories for valued products. Algal Res..

[31] Monte J., Sá M., Galinha C.F., Costa L., Hoekstrad H., Brazinha C., Crespo J.G. (2018). Harvesting of Dunaliella salina by membrane filtration at pilot scale. Sep. Purif. Technol..

[32] Desmorieux H., Hernandez F. Biochemical and physical criteria of Spirulina after different drying processes. Proceedings of the 14th International Drying Symposium (IDS).

[33] Walsh M.J., Van Doren L.G., Shete N., Prakash A., Salim U. (2018). Financial tradeoffs of energy and food uses of algal biomass under stochastic conditions. Appl. Energy.

[34] Nova P., Martins A.P., Teixeira C., Abreu H., Silva J.G., Machado Silva A., Freitas A.C., Gomes A.M. (2020). Foods with microalgae and seaweeds fostering consumers health: A review on scientific and market innovations. J. Appl. Phycol..

[35] Enzing C., Ploeg M., Barbosa M., Sijtsma L., Vigani M., Parisi C., Cerezo E.R. (2014). Microalgae-based products for the food and feed sector: An outlook for Europe. EUR—Scientific and Technical Research Series.

[36] Amorim M.L., Soares J., dos Reis Coimbra J.S., de Oliveira Leite M., Teixeira Albino L.F., Arêdes Martins M. (2020). Microalgae proteins: Production, separation, isolation, quantification, and application in food and feed. Crit. Rev. Food Sci. Nutr..

[37] Grossman A. (2016). Nutrient acquisition: The generation of bioactive vitamin B12 by microalgae. Curr. Biol..

[38] Han W., Clarke W., Pratt S. (2016). Cycling of iodine by microalgae: Iodine uptake and release by a microalgae biofilm in a groundwater holding pond. Ecol. Engin..

[39] Iwamoto K., Shiraiwa Y. (2012). Characterization of intracellular iodine accumulation by iodine-tolerant microalgae. Procedia Environm. Sci..

[40] Müssig K., Preedy V.R., Burrow G.N., Watson R. (2009). Chapter 93—Iodine-induced toxic effects due to seaweed consumption. Comprehensive Handbook of Iodine.

[41] Begum H., Yusoff F.M.D., Banerjee S., Khatoon H., Shariff M. (2016). Availability and utilization of pigments from microalgae. Crit. Rev. Food Sci. Nutr..

[42] Kartik A., Akhil D., Lakshmi D., Gopinath K.P., Arun J., Sivaramakrishnan R., Pugazhendhi A. (2021). A critical review on production of biopolymers from algae biomass and their applications. Biores. Technol..

[43] Chacon-Lee T.L., Gonzalez-Marino G.E. (2010). Microalgae for “healthy” foods—Possibilities and challenges. Compr. Rev. Food Sci. Food Saf..

[44] FAO/WHO (1973). Energy and protein requirement. Report of a Joint FAO/WHO Ad Hoc Expert Committee.

[45] Becker E.W. (2007). Micro-algae as a source of protein. Biotechnol. Adv..

[46] Sandgruber F., Gielsdorf A., Baur A.C., Schenz B., Müller S.M., Schwerdtle T., Stangl G.I., Griehl C., Lorkowski S., Dawczynski C. (2021). Variability in macro- and micronutrients of 15 commercially available microalgae powders. Mar. Drugs.

[47] Lafarga T. (2019). Effect of microalgal biomass incorporation into foods: Nutritional and sensorial attributes of the end products. Algal Res..

[48] da Silva S.P., Ferreira do Valle A., Perrone D. (2021). Microencapsulated Spirulina maxima biomass as an ingredient for the production of nutritionally enriched and sensorially well-accepted vegan biscuits. LWT Food Sci. Technol..

[49] Mantecón L., Moyano R., Cameánc A.M., Jos A. (2019). Safety assessment of a lyophilized biomass of Tetraselmis chuii (TetraSOD^®^) in a 90 day feeding study. Food Chem. Toxicol..

[50] Plankton Marino. https://www.planctonmarino.com.

[51] Bernaerts T.M.M., Gheysen L., Foubert I., Hendrickx M.E., Van Loey A.M. (2019). The potential of microalgae and their biopolymers as structuring ingredients in food: A review. Biotechnol. Adv..

[52] Sahni P., Sharma S., Singh B. (2019). Evaluation and quality assessment of defatted microalgae meal of Chlorella as an alternative food ingredient in cookies. Nutr. Food Sci..

[53] Onacik-Gür S., Zbikowska A., Majewska B. (2017). Effect of Spirulina (Spirulina platensis) addition on textural and quality properties of cookies. Ital. J. Food Sci..

[54] Kumoro A.C., Johnny D., Alfilovita D. (2016). Incorporation of microalgae and seaweed in instant fried wheat noodles manufacturing: Nutrition and culinary properties study. Int. Food Res. J..

[55] de Marco E.R., Steffolani M.E., Martínez C.S., León C.S. (2014). Effects of spirulina biomass on the technological and nutritional quality of bread wheat pasta. LWT Food Sci. Technol..

[56] El-Baz F.K., Abdo S.M., Hussein A.M.S. (2017). Microalgae Dunaliella salina for use as food supplement to improve pasta quality. Int. J. Pharm. Sci. Rev. Res..

[57] Fradique M., Batista A.P., Nunes M.C., Gouveia L., Bandarra N.M., Raymundo A. (2010). Incorporation of Chlorella vulgaris and Spirulina maxima biomass in pasta products. Part 1: Preparation and evaluation. J. Sci. Food Agric..

[58] Buono S., Langellotti A.L., Martello A., Rinna F., Fogliano V. (2014). Functional ingredients from microalgae. Food Funct..

[59] Fradique M., Batista A.P., Nunes M.C., Gouveia L., Bandarra N.M., Raymundo A. (2013). Isochrysis galbana and Diacronema vlkianum biomass incorporation in pasta products as PUFA’s source. LWT Food Sci. Technol..

[60] Böhm T., Berger H., Nejabat M., Riegler T., Kellner F., Kuttke M., Sagmeister S., Bazanella M., Stolze K., Daryabeigi A. (2013). Food-derived peroxidized fatty acids may trigger hepatic inflammation: A novel hypothesis to explain steatohepatitis. J. Hepatol..

[61] Ryckebosch E., Bruneel C., Termote-Verhalle R., Goiris K., Muylaert K., Foubert I. (2014). Nutritional evaluation of microalgae oils rich in omega-3 long chain polyunsaturated fatty acids as an alternative for fish oil. Food Chem..

[62] Lemahieu C., Bruneel C., Ryckebosch E., Muylaert K., Buyse J., Foubert I. (2015). Impact of different omega-3 polyunsaturated fatty acid (n-3 PUFA) sources (flaxseed, Isochrysis galbana, fish oil and DHA Gold) on n-3 LC-PUFA enrichment (efficiency) in the egg yolk. J. Funct. Foods.

[63] Shen Y., Guo C., Lu T., Ding X.-Y., Zhao M.-T., Zhang M., Liu H.-L., Song L., Zhou D.-Y. (2021). Effects of gallic acid alkyl esters and their combinations with other antioxidants on oxidative stability of DHA algae oil. Food Res. Int..

[64] de Jesus Raposo M.F., de Morais A.M.M.B., de Morais R.M.S.C. (2015). Carotenoids from marine microalgae: A valuable natural source for the prevention of chronic diseases. Mar. Drugs.

[65] Turck D., Castenmiller J., de Henauw S., Hirsch-Ernst K.I., Kearney J., Maciuk A., Mangelsdorf I., McArdle H.J., Naska A., EFSA NDA Panel (EFSA Panel on Nutrition, Novel Foods and Food Allergens) (2020). Scientifc Opinion on the safety of astaxanthin for its use as a novel food in food supplements. EFSA J..

[66] Turck D., EFSA NDA Panel (EFSA Panel on Nutrition, Novel Foods and Food Allergens), EFSA Panel on Food Additives and Nutrient Sources added to Food (ANS) (2012). Scientific opinion on the re-evaluation of mixed carotenes (E 160a(i)) and beta-carotene (E 160a (ii)) as a food additive. EFSA J..

[67] Pereira A.G., Otero P., Echave J., Carreira-Casais A., Chamorro F., Collazo N., Jaboui A., Lourenço-Lopes C., Simal-Gandara J., Prieto M.A. (2021). Xanthophylls from the sea: Algae as source of bioactive carotenoids. Mar. Drugs.

[68] Fen L., Nie K., Jiang H., Fan W. (2019). Effects of lutein supplementation in age-related macular degeneration. PLoS ONE.

[69] Plaza M., Cifuentes A., Ibanez E. (2008). In the search of new functional food ingredients from algae. Trends Food Sci. Technol..

[70] Matos J., Cardoso C., Bandarra N.M., Afonso C. (2017). Microalgae as healthy ingredients for functional food: A review. Food Funct..

[71] Boussiba S., Vonshak A. (1991). Astaxanthin accumulation in the green alga Haematococcus pluvialis. Plant Cell Physiol..

[72] Heussner A.H., Mazija L., Fastner J., Dietrich D.R. (2012). Toxin content and cytotoxicity of algal dietary supplements. Toxicol. Appl. Pharmacol..

[73] García J.L., de Vicente M., Galán B. (2017). Microalgae, old sustainable food and fashion nutraceuticals. Microb. Biotechnol..

[74] Eltanahy E., Torky A. (2021). Microalgae as cell factories: Food and feed-grade high-value metabolites. Microalgal Biotechnology: Recent Advances, Market Potential, and Sustainability.

